# Supplementary material for: Nutritional deficiency induces nucleus pulposus cell apoptosis via the ATF4-PKM2-AKT signal ﻿axis
Source: BMC Musculoskelet Disord. 2022 Nov 2;23:946. doi: 10.1186/s12891-022-05853-1 (PMC9628105; doi:10.1186/s12891-022-05853-1)
Supplement: Supplementary file 1 — Supplementary Material 1 [file 12891_2022_5853_MOESM1_ESM.docx]

**FNutritional deficiency induces nucleus pulposus cell apoptosis via the ATF4-PKM2-AKT signal axis**

Ningfeng Zhou^1#^, Bin Shen^1#^, Chong Bai^1#^, Liang Ma^1^, Shanjin Wang^1*^, Desheng Wu^1*^

^1^Department of Spinal Surgery, Shanghai East Hospital, Tongji University School of Medicine, Shanghai, China.

^#^ Ningfeng Zhou, Bin Shen and Chong Bai are equally contributed to this work.

*Corresponding author:

Dr. Desheng Wu, eastspinesci@163.com, Department of Spinal Surgery, Shanghai East Hospital, Tongji University School of Medicine, Shanghai, China.

Dr. Shanjin Wang, kingspine@163.com, Department of Spinal Surgery, Shanghai East Hospital, Tongji University School of Medicine, Shanghai, China.

The authors declare no conflict of interest.

**Abstract**

The intervertebral disc is the largest avascular tissue in the human body. The nucleus pulposus (NP) consumes glucose and oxygen to generate energy to maintain cellular metabolism via nutrients that diffuse from the cartilage endplate. The microenvironment in the intervertebral disc becomes nutritionally deficient during degeneration, and nutritional deficiency has been shown to inhibit the viability and proliferation of NP cells. To investigate the molecular mechanism by which nutritional deficiency reduces viability and decreases proliferation, we created an *in vitro* model by using decreasing serum concentration percentages. In this study, we found that nutritional deficiency reduced NP cell viability and increased cell apoptosis and that the upregulation of ATF4 expression and the downregulation of PKM2 expression were involved in this process. Moreover, we found that PKM2 inhibition can reduce the cell apoptosis induced by ATF4 silence under nutritional deficiency. We further analyzed the mechanism by which nutritional deficiency reduces NP cell viability and increases cell apoptosis. Our findings revealed that PKM2 inhibition reduces the cell apoptosis induced by ATF4 silence under nutritional deficiency by inhibiting AKT phosphate. Revealing the function and mechanism of NP cell development under nutritional deficiency will provide new insights into the etiology, diagnosis, and treatment of intervertebral disc and related diseases.

**Keywords:** intervertebral disc degeneration; nucleus pulposus; nutritional deficiency; ATF4; PKM2

**Introduction**

Intervertebral disc degeneration (IVDD) is considered the leading cause of low back pain, which is an extremely common musculoskeletal disorder that affects people of all ages and results in global disability [1, 2]. The annual cost of IVDD treatments is over $100 billion in the United States alone, which is even more than the total cost of treating stroke, respiratory infection, diabetes, coronary artery disease, and rheumatoid disease [3-5]. The currently available treatments provide only symptomatic relief from pain through injections, physical therapy, and activity modification or surgical intervention, such as open microdiscectomy or percutaneous endoscopic lumbar discectomy [3, 6-10]. These interventions cannot prevent the progression of degeneration or restore the physiological function of the intervertebral disc (IVD), and understanding the exact etiology is essential for curing or preventing the progression of IVDD. The IVD is composed of the central nucleus pulposus (NP), the surrounding annulus fibrosus (AF) ring, and cartilaginous endplates. Nucleus pulposus cells (NPCs) are the major cells that reside in the NP and are important for the preservation of the extracellular matrix (ECM), such as aggrecan and type II collagen (Col2α1), and thus maintain the normal physiological function of IVDs [11-13]. Although the exact etiology of IVDD remains unclear, significantly increased NPC apoptosis and reduced cell proliferation are proven to be important contributors to IVDD [11, 14, 15].

As the largest avascular organ in our body, the IVD was reported to obtain all essential nutrients through the cartilage endplate [16]. The main energy supply of IVD cells is provided by glycolysis, which consumes glucose and produces lactic acid at a relatively high rate [17]. An adequate level of glucose is essential for maintaining the viability of IVD cells. If the glucose concentration is lower than 0.5 mmol/L for more than a few days, the cells will begin to die. In addition, a low pH (<6.4), caused mainly by lactic acid accumulation, will also affect the viability of IVD cells [18, 19]. Gradients of oxygen, glucose, and lactic acid exist throughout the disc. Oxygen and glucose concentrations fall and lactic acid concentrations rise toward the center of the nucleus; hence, the center of the disc has a low glucose and oxygen concentration and is acidic [20]. It was found that in the center, the concentration of nutrients ranges from 1% to 5% depending on the oxygen level [21]. With aging and degeneration in the IVD, the supply of nutrients, such as oxygen, glucose, and serum, reduces significantly, leading to a microenvironment with severe nutritional deficiency, which causes metabolic disturbances, induces apoptosis, and reduces the proliferation of NPCs [22-24].

Tetrameric pyruvate kinase (PK) catalyzes the final step in glycolysis, converting phosphoenolpyruvate into pyruvate. Transcripts from the PKM locus are alternatively spliced into two major isoforms, M1 and M2 [25, 26]. Pyruvate kinase M2 (PKM2) can be aggregated into tetrameric and dimeric forms. PKM2 in the dimer state can enter the nucleus to regulate gene expression and phosphorylation, induce acetylation and other modifications, mediate the different intracellular localizations of PKM2, and play an important role in cell energy supply and cell proliferation [26, 27].

Activating transcription factor 4 (ATF4) is a member of the ATF/CREB family and plays an important role in amino acid and glucose metabolism, the intracellular anti-oxidative stress response, and the transcriptional regulation of inflammatory factors. ATF4 overexpression was reported to trigger a cascade of reactive oxygen species (ROS) and to induce cell apoptosis [28, 29]. Conversely, the deletion of ATF4 can significantly inhibit ROS production and apoptotic processes during physiological and pathological conditions [30]. The ATF4 transcription factor can function together with Runx2 and osterix and plays a role in osteoblast differentiation [31-33]. The phosphatidylinositol 3-kinase (PI3K)/Akt signaling pathway is the key to cell proliferation, apoptosis, and differentiation in various tissues and cell types [34]. Smith et al. [35] found that the Akt signal is crucial for the acute stimulation of the mitochondrial oxygen consumption rate (OCR) during the process of Wnt- and BMP-mediated osteoinduction. Some studies have shown that cyclic stretch promoted the energy metabolism in osteoblast-like MG-63 cells by regulating glucose consumption, lactate levels, ATP levels, and energy metabolism-related enzymes partially through the Akt/mTOR/p70s6k signaling pathway [36]. Considering the close relationship between PKM2, ATF4, cell apoptosis, cell proliferation, and energy metabolism, we investigated NPC apoptosis induced by nutritional deficiency via the ATF4-PKM2-AKT signal axis.

In this study, we found that nutritional deficiency reduced NP cell viability and increased cell apoptosis and that the expression of ATF4 and PKM2 was involved in this process. In addition, we found that PKM2 inhibition can reduce the cell apoptosis induced by ATF4 silence under nutritional deficiency. We further analyzed the mechanism by which nutritional deficiency reduces NP cell viability and increases cell apoptosis. Our findings revealed that PKM2 inhibition reduced the cell apoptosis induced by ATF4 silence under nutritional deficiency by inhibiting AKT phosphate.

**Results**

**Nutritional deficiency can reduce cell viability and the expression of ATF4 and inhibit the expression of PKM2 in NPCs**

To test how cell viability was affected by nutritional deficiency, we performed an *in vitro* colorimetric cell counting kit-8 (CCK8) cell viability assay in primary NPCs. A cell nutritional deficiency model was created by using decreasing serum concentration percentages. The results showed that cell viability significantly reduced from control cells to 5%, 1%, and 0% serum-containing media, indicating that nutritional deficiency can reduce cell viability (Figure 1A). Moreover, our western blot results showed that the expression of ATF4 was increased, while that of PKM2 was decreased with decreasing concentration of serum-containing media (Figure 1B, C). We used 1% serum-containing media to stimulate NPCs for different time courses, and the levels of ATF4 and PKM2 were determined by western blotting. The expression of ATF4 was increased, while that of PKM2 was decreased in a time-dependent manner, as detected by western blotting (Figure 1D, E). Therefore, these studies showed that nutritional deficiency can reduce cell viability and the expression of ATF4 and inhibit the expression of PKM2 in NPCs.

**ATF4 silence can induce NP cells’ apoptosis under nutritional deficiency**

To further investigate the role of the ATF4 gene in NPCs under nutritional deficiency, we used the siRNA-induced gene knockout method to knock out the ATF4 gene in NPCs. The gene knockout effect was confirmed by RT-qPCR and western blotting (Figure 2A-C). The CCK8 assay was used to investigate how the ATF4 gene knockout affected cell viability under nutritional deficiency by using 1% serum-containing media (Figure 2D). The CCK8 assay suggested that the ATF4 gene knockout can reduce cell viability under nutritional deficiency by using 1% serum-containing media. Flow cytometry was used to investigate how the ATF4 gene knockout affected cell apoptosis under nutritional deficiency (Figure 2E, F). The results suggested the ATF4 gene knockout can increase cell apoptosis under nutritional deficiency by using 1% serum-containing media. To further confirm the apoptosis induced by the ATF4 gene knockout under nutritional deficiency, the apoptosis proteins Bax, Bcl2, and cleaved caspase-3 were detected by a western blot assay. As shown in Figures 2G and 2H, cell apoptosis was increased under nutritional deficiency.

**ATF4 silence can increase NP cells’ glucose uptake and lactic acid level under nutritional deficiency**

Glucose uptake and the lactic acid level can reflect the cell metabolism level and a healthy condition. As expected, the glucose uptake level was significantly reduced in the group with 1% serum-containing media (Figure 3A). However, the glucose uptake level increased after knocking out the ATF4 gene. The lactic acid level decreased from 9.8 mmol/L in the control group to 2.1 mmol/L in the 1% serum-containing media and increased to 5.8 mmol/L after knocking out the ATF4 gene (Figure 3B). Moreover, our western blot results showed that the expression of ATF4 and HIF-1α was increased, while that of PKM2 was decreased in 1% serum-containing media, as compared with the control. Interestingly, compared with the expression in the group with 1% serum-containing media, the expression of PKM2 and HIF-1α was increased when the ATF4 gene was silenced (Figure 3C, D). The apoptosis caused by nutritional deficiency may be brought about by affecting ATF4- or PKM2-induced cell energy metabolism.

Considering that AKT is involved in cell proliferation, apoptosis, and energy metabolism signaling, we detected the AKT and P-AKT expression levels in each group. Our western blot results showed that the expression of P-AKT decreased in 1% serum-containing media and increased in the ATF4 gene knockout group (Figure 4A, B). These data suggested that ATF4 silence can induce NP cells’ apoptosis under nutritional deficiency via promoting AKT phosphate.

**PKM2 inhibition can reduce the cell apoptosis induced by ATF4 silence under nutritional deficiency**

To test how cell viability was affected by PKM2 inhibition induced by ATF4 silence under nutritional deficiency, we performed an *in vitro* colorimetric CCK8 cell viability assay in primary NPCs. The results revealed that PKM2 inhibition can increase cell viability under nutritional deficiency. However, PKM2 inhibition can decrease cell viability after ATF4 silence under nutritional deficiency (Figure 5A). The flow cytometry results demonstrated that PKM2 inhibition can decrease cell apoptosis under nutritional deficiency. However, PKM2 inhibition can increase cell apoptosis after ATF4 silence under nutritional deficiency (Figure 5B, C).

**PKM2 inhibition can reduce NP cells’ glucose uptake and lactic acid level induced by ATF4 silence under nutritional deficiency**

The cells’ glucose uptake and lactic acid level were tested to show how PKM2 affected the cells’ energy metabolism. The cells’ glucose uptake and lactic acid level were significantly decreased in the PKM2 inhibition group under nutritional deficiency. What is more, PKM2 inhibition can reduce the NP cells’ glucose uptake and lactic acid level induced by ATF4 silence under nutritional deficiency, indicating that PKM2 is downstream of ATF4 (Figure 6A, B). Consistent with this, HIF-1α was reduced in the PKM2 inhibition group under nutritional deficiency, and PKM2 inhibition can reduce the HIF-1a level induced by ATF4 silence under nutritional deficiency (Figure 6C, D).

**PKM2 inhibition reduces the cell apoptosis induced by ATF4 silence under nutritional deficiency by inhibiting AKT phosphate**

To further confirm the apoptosis induced by PKM2 inhibition under nutritional deficiency, the apoptosis proteins Bax, Bcl2, and cleaved caspase-3 were detected by a western blot assay. As shown in Figure 7A, B, cell apoptosis was decreased by PKM2 inhibition under nutritional deficiency. However, PKM2 inhibition can increase cell apoptosis after ATF4 silence under nutritional deficiency.

Our western blot results showed that the expression of P-AKT was decreased by PKM2 inhibition under nutritional deficiency (Figure 7C, D). KM2 inhibition can increase AKT phosphate after ATF4 silence under nutritional deficiency.

**Methods and materials**

**Human NPC culture**

Human NPCs were dissected during surgical disc procedures performed on patients with lumbar burst fractures. All the methods were performed in accordance with the guidelines and regulations of Shanghai East Hospital. All the participants consented to participate in this study. NPCs were isolated and cultured as previously described [37]. After isolation, NPCs were re-suspended in Dulbecco’s modified Eagle’s medium/F-12 (HyClone, USA) containing 10% fetal bovine serum (Gibco, USA) and 50 units/mL of penicillin and streptomycin (HyClone, USA), and then incubated at 37 °C in a humidified atmosphere with 95% air and 5% CO_2_. The confluent cells were detached by trypsinization and then seeded into a cell culture dish of 10 cm in a complete culture medium for passing. The medium was changed every other day during culturing. When it reached 90% confluency, third passage human NPCs were cultured in different percentages of serum-containing media as required for the subsequent experiments [37].

**Glucose uptake assay**

After transfection or culturing under nutritional deficiency, NPCs were washed twice with Krebs-Ringer-Phosphate-HEPES (KRPH) buffer and starved of glucose by incubation with KRPH buffer containing 0.2% bovine serum albumin (BSA) at 37 °C for 40 min. The NPCs were then stimulated with 100 nM insulin (Sigma-Aldrich) for 30 min in KRPH buffer supplemented with 0.2% BSA. Glucose transport was determined by subsequent stimulation with 2-deoxy-D-glucose-6-phosphate (2DG6P) at a final concentration of 0.1 mM for 20 min. The reaction was terminated by washing the cells four times with ice-cold PBS. The cells were lysed in lysis buffer, and glucose uptake was assessed using the glucose uptake assay kit (K682-50, Biovision) in accordance with the manufacturer's instructions. The absorbance was measured at a wavelength of 412 nm on a microplate reader.

**L-lactate concentration detection**

NPCs were cultured in normoxia or hypoxia after transfection with siRNA or cultured under nutritional deficiency. The supernatant was collected after 24 h and deproteinized using a 10KD filter (EMD Millipore, Billerica, MA, USA) by centrifuging for 30 min at room temperature. L-lactate was quantified using an assay kit (A019-2, Nanjing Jiancheng) using the manufacturer’s protocol.

**Western blot**

The expression levels of proteins were determined by western blot analysis of the total protein extracts from NPCs. Cell samples were lysed in RIPA buffer and sonicated, and the protein concentrations were calculated using the BCA protein assay kit. Proteins were loaded onto 8% SDS-PAGE gels and transferred to PVDF membranes (Millipore, Billerica, MA, USA). After blocking for 1 h, the membranes were incubated with primary antibodies overnight at 4 °C. Primary antibodies specific to ATF4 (1:1000, 10835-1-AP, Proteintech), PKM2 (1:1000, 15822-1-AP, Proteintech), Bax (1:1000, Ab53154, Abcam), Bcl2 (1:1000, Ab196495, Abcam), cleaved caspase-3 (1:1000, Ab2302，Abcam), and GADPH (1:1000, 5174, CST) were used. Negative controls were performed with normal rabbit IgG (Sigma) under the same conditions. After washing with Tris Buffered Saline with Tween (TBST) three times, the membranes were incubated with the respective secondary antibodies. Then the bands were detected with ECL plus reagent (Millipore) by the ChemiDoc™ XRS + System (BIO-RAD, USA). Relative expression levels of proteins were determined by quantitative densitometric analysis using image analysis software (Image lab, Bio-Rad, USA).

**Flow cytometry**

Cells were resuspended in 100 mL of cell staining buffer, incubated with antibodies for 20 min on ice to block Fc receptors, and stained with fluorochrome-conjugated or isotype control antibodies on ice for 20 min assessed using a glucose uptake assay kit (K682-50, Biovision) in accordance with flow cytometry (C1062, Beyond). The acquired raw data were further analyzed using FlowJo software (Tree Star). Representative plots of at least three independent biological samples are shown in Figure.

**Cell Counting Kit-8 (CCK8) assay**

Human NPCs were seeded into 96-well plates and incubated in the presence of decreased concentrations of serum (10%, 5%, 1%, 0%) for 0 h, 24 h, 48 h, and 72 h. Cell survival rate was determined using the established CCK-8 assay. Each well was incubated with a CCK-8 solution (CP002, SAB) for 4 h, and the absorbance was measured at 590 nm using a spectrophotometer. Wells containing only medium served as blank controls.

**Statistics**

Student’s t-test for the comparison between two groups or a one-way ANOVA followed by Tukey’s multiple comparison test for grouped samples was performed. The program GraphPad Prism (GraphPad Software, Inc., San Diego, USA) was used for these analyses. P < 0.05 was considered to be significant. NS denotes not significant.

**Discussion**

The NP is the largest avascular tissue in the human body, and nutrients diffuse across the cartilage endplate to reach the intervertebral cells and disc cells, thus consuming glucose and oxygen to generate energy to maintain cellular metabolism [22]. The microenvironment becomes nutritionally deficient during degeneration. Nutritional deficiency has been shown to inhibit the viability and proliferation of NPCs [22, 23, 38]. To investigate the mechanism by which nutritional deficiency reduces NPC viability and proliferation, we created an *in vitro* model by using decreasing serum concentration percentages. In agreement with previous studies, our study suggested that nutritional deficiency can inhibit the viability and proliferation of NPCs [39]. Then, we found a new mechanism by which nutritional deficiency can induce NPC apoptosis. Namely, PKM2 inhibition reduces the cell apoptosis induced by ATF4 silence under nutritional deficiency via inhibiting AKT phosphate.

It has been reported that ATF4 increased in NPCs in the early stages of glucose deprivation and that ATF4 siRNA inhibited both ROS production and apoptosis [40]. Zong et al. [28] reported that upregulating ATF4 expression could increase cellular ROS and the sensitivity to apoptosis. ATF4 is upregu­lated by stress signals, including oxidative stress, hypoxia, and endoplasmic reticulum stress. ATF4 also affects the expression of genes involved in oxidative stress [29, 31, 41]. ATF4 gene expression was silenced resulting in the downregulation of both CHOP and caspase‑9, reversing the negative effect. In agreement with these previous observations, we found that ATF4 expression was increased under severe nutritional deficiency in NPCs and that ATF4 deletion can increase cell viability and proliferation and reduce cell apoptosis. We also found that the expression of ATF4 decreased with higher glucose uptake and LD levels in NPCs. Consistent with this, ATF4 was reported to be closely related to thermogenesis and amino acid, glucose, and lipid metabolism, with ATF4 mutant mice having increased energy expenditure [32, 33].

The role of PKM2 in glucose metabolism has been well established [27, 42-44]. The effect of PKM2 is currently considered to be the interception of glucose metabolism, whereby the metabolic pathway is transformed into the pentose phosphate pathway, the uronic acid pathway, the polyol pathway, etc. for the synthesis of five-carbon ribose and non-essential amino acids. Amino acids, fatty acids, glucose intermediates, and bypass metabolites can regulate PKM2 enzyme composition and enzyme activity [26]. Consistent with this, we found that PKM2 was significantly reduced with decreased glucose uptake during nutritional deficiency in NPCs. PKM2 recently also received much attention for its noncanonical roles in tumorigenesis, functioning as a dimer enhancing the transcriptional activity of Oct-4, b-catenin, and HIF-1α [42, 43, 45]. Previous studies demonstrated that PKM2 interacts directly with the HIF-1α subunit and promotes the transactivation of HIF-1 target genes [42, 43, 46]. However, HIF-1a activity in AF cells was unaffected by PKM2 overexpression and tetrameric stabilization. Unlike that in other cell types, HIF-1 activity in NPCs is not influenced by manipulation of PKM2 and JMJD5 levels because of NPCs’ physiologically hypoxic niche [45]. In our study, we found that the expression level of HIF-1 was negatively related to the expression of HIF under nutritional deficiency. The regulation mechanism is still unclear, and it needs to be further investigated in the future.

PI3Ks are a unique family of intracellular lipid kinases, and Akt is a serine/threonine kinase. Once activated by different agents, PI3Ks change phosphatidylinositol 4,5-biphosphate (PIP2) into phosphatidylinositol 3,4,5-triphosphate (PIP3) and further activate Akt. Activated Akt modulates biological processes, including cell proliferation and apoptosis, through interaction with downstream proteins. More importantly, Akt activation has been shown to have a protective effect on IVD degeneration. Activation of Akt signaling increases Sox9 expression and activity, which induces the expression of aggrecan in NPCs [47]. Insulin-like growth factor-1 (IGF-1) promotes IVD cell proliferation by activating this pathway [48]. Many adverse factors contribute to IVD degeneration, such as an adverse microenvironment and cytokines. Previous studies indicated that activation of AKT signaling attenuated NPC apoptosis induced by high-magnitude compression or hyperosmotic conditions [49]. In addition, AKT could protect NPCs against apoptosis induced by some cytokines, such as IL-1β and TNF-α [50].

The relation between ATF4 and PKM2 has been investigated in several studies [44, 51, 52]. This is the first study to investigate the relationship between ATF4 and PKM2 in NPC apoptosis under nutritional deficiency. The PKM2 mutation has been found in many tumors, and its deletion can increase cell apoptosis [25, 27, 53, 54]. In our study, we find that the overexpression of PKM2 can reduce NPC apoptosis under nutritional deficiency. Interestingly, the overexpression of PKM2 in NPCs with an ATF4 mutation can reduce cell apoptosis and reduce the phosphatidylinositol-AKT induced by the ATF4 mutation under nutritional deficiency. This suggests that PKM2 may function downstream of ATF4 during NPC apoptosis induced by nutritional deficiency.

**Funding**

Research reported in this publication was supported by the National Natural Science Foundation of China (No. 81972106), Shanghai Natural Science Foundation (No. 19ZR1441700; Shanghai, China), and Discipline Leader Training Project of Pudong New Area Municipal Health Commission (No. PWRd2020-03; Shanghai, China)

**Acknowledgements**

The authors thank you the funding from National Natural Science Foundation of China (No. 81972106), Shanghai Natural Science Foundation (No. 19ZR1441700; Shanghai, China), and Discipline Leader Training Project of Pudong New Area Municipal Health Commission (No. PWRd2020-03; Shanghai, China)

**Author contributions**

Ningfeng Zhou, Bin Shen, Chong Bai and Liang Ma, performed the experiments, interpreted the data and wrote the initial draft of the manuscript. Shanjin Wang and Desheng Wu conceived, supervised the study and wrote the manuscript.

**Conflict of interest**

The authors declare no conflict of interest.

**Ethical Approval**

Experimental study of IVD specimens were approved prospectively by the authors’ human subjects Institutional Review Board. (Institutional Review Board of Shanghai east hospital).

**Informed consent**

Informed consent was obtained from all subjects and/or their legal guardian(s) in this study.

**Consent for publication**

N/A.

**Availability of data and materials**

The datasets used and/or analysed during the current study available from the corresponding author on reasonable request.

**References**

1. Moriguchi Y, Alimi M, Khair T, Manolarakis G, Berlin C, Bonassar LJ, Hartl R: **Biological Treatment Approaches for Degenerative Disk Disease: A Literature Review of In Vivo Animal and Clinical Data**. *Global Spine J* 2016, **6**(5):497-518.

2. Chen F, Wu T, Bai C, Guo S, Huang W, Pan Y, Zhang H, Wu D, Fu Q, Chen Q *et al*: **Serum apolipoprotein B/apolipoprotein A1 ratio in relation to intervertebral disk herniation: a cross-sectional frequency-matched case-control study**. *Lipids in health and disease* 2021, **20**(1):79.

3. Miller JA, Schmatz C, Schultz AB: **Lumbar disc degeneration: correlation with age, sex, and spine level in 600 autopsy specimens**. *Spine (Phila Pa 1976)* 1988, **13**(2):173-178.

4. Adams MA, Roughley PJ: **What is intervertebral disc degeneration, and what causes it?** *Spine (Phila Pa 1976)* 2006, **31**(18):2151-2161.

5. Vincent K, Mohanty S, Pinelli R, Bonavita R, Pricop P, Albert TJ, Dahia CL: **Aging of mouse intervertebral disc and association with back pain**. *Bone* 2019, **123**:246-259.

6. Li X, Han Y, Cui J, Yuan P, Di Z, Li L: **Efficacy of Warm Needle Moxibustion on Lumbar Disc Herniation: A Meta-Analysis**. *Journal of evidence-based complementary & alternative medicine* 2016, **21**(4):311-319.

7. Li X, Han Y, Di Z, Cui J, Pan J, Yang M, Sun G, Tan J, Li L: **Percutaneous endoscopic lumbar discectomy for lumbar disc herniation**. *J Clin Neurosci* 2016, **33**:19-27.

8. Li X, Hu Z, Cui J, Han Y, Pan J, Yang M, Tan J, Sun G, Li L: **Percutaneous endoscopic lumbar discectomy for recurrent lumbar disc herniation**. *Int J Surg* 2016, **27**:8-16.

9. Smith LJ, Chiaro JA, Nerurkar NL, Cortes DH, Horava SD, Hebela NM, Mauck RL, Dodge GR, Elliott DM: **Nucleus pulposus cells synthesize a functional extracellular matrix and respond to inflammatory cytokine challenge following long-term agarose culture**. *Eur Cell Mater* 2011, **22**:291-301.

10. Zheng L, Cao Y, Ni S, Qi H, Ling Z, Xu X, Zou X, Wu T, Deng R, Hu B *et al*: **Ciliary parathyroid hormone signaling activates transforming growth factor-beta to maintain intervertebral disc homeostasis during aging**. *Bone research* 2018, **6**:21.

11. Hickman TT, Rathan-Kumar S, Peck SH: **Development, Pathogenesis, and Regeneration of the Intervertebral Disc: Current and Future Insights Spanning Traditional to Omics Methods**. *Front Cell Dev Biol* 2022, **10**:841831.

12. Li X, Yang S, Qin L, Yang S: **Type II collagen-positive embryonic progenitors are the major contributors to spine and intervertebral disc development and repair**. *Stem Cells Transl Med* 2021, **10**(10):1419-1432.

13. Li X, Yang S, Yuan G, Jing D, Qin L, Zhao H, Yang S: **Type II collagen-positive progenitors are important stem cells in controlling skeletal development and vascular formation**. *Bone Res* 2022, **10**(1):46.

14. Ohnishi T, Iwasaki N, Sudo H: **Causes of and Molecular Targets for the Treatment of Intervertebral Disc Degeneration: A Review**. *Cells* 2022, **11**(3).

15. Zhang C, Peng X, Wang F, Xie Z, Chen L, Wu X: **Update on the Correlation Between Mitochondrial Dysfunction and Intervertebral Disk Degeneration**. *DNA and cell biology* 2022, **41**(3):257-261.

16. Han Y, Li X, Yan M, Yang M, Wang S, Pan J, Li L, Tan J: **Oxidative damage induces apoptosis and promotes calcification in disc cartilage endplate cell through ROS/MAPK/NF-κB pathway: Implications for disc degeneration**. *Biochemical and biophysical research communications* 2019, **516**(3):1026-1032.

17. Holm S, Maroudas A, Urban JP, Selstam G, Nachemson A: **Nutrition of the intervertebral disc: solute transport and metabolism**. *Connective tissue research* 1981, **8**(2):101-119.

18. Horner HA, Urban JP: **2001 Volvo Award Winner in Basic Science Studies: Effect of nutrient supply on the viability of cells from the nucleus pulposus of the intervertebral disc**. *Spine* 2001, **26**(23):2543-2549.

19. Bibby SR, Jones DA, Ripley RM, Urban JP: **Metabolism of the intervertebral disc: effects of low levels of oxygen, glucose, and pH on rates of energy metabolism of bovine nucleus pulposus cells**. *Spine* 2005, **30**(5):487-496.

20. Urban JP, Smith S, Fairbank JC: **Nutrition of the intervertebral disc**. *Spine* 2004, **29**(23):2700-2709.

21. Dolor A, Sampson SL, Lazar AA, Lotz JC, Szoka FC, Fields AJ: **Matrix modification for enhancing the transport properties of the human cartilage endplate to improve disc nutrition**. *PloS one* 2019, **14**(4):e0215218.

22. Rinkler C, Heuer F, Pedro MT, Mauer UM, Ignatius A, Neidlinger-Wilke C: **Influence of low glucose supply on the regulation of gene expression by nucleus pulposus cells and their responsiveness to mechanical loading**. *Journal of neurosurgery Spine* 2010, **13**(4):535-542.

23. Jünger S, Gantenbein-Ritter B, Lezuo P, Alini M, Ferguson SJ, Ito K: **Effect of limited nutrition on in situ intervertebral disc cells under simulated-physiological loading**. *Spine* 2009, **34**(12):1264-1271.

24. Lang G, Liu Y, Geries J, Zhou Z, Kubosch D, Südkamp N, Richards RG, Alini M, Grad S, Li Z: **An intervertebral disc whole organ culture system to investigate proinflammatory and degenerative disc disease condition**. *Journal of tissue engineering and regenerative medicine* 2018, **12**(4):e2051-e2061.

25. Park JH, Lee JS, Oh Y, Lee JS, Park HE, Lee H, Park YS, Kyung SY, Kim HS, Yoon S: **PKM2 Is Overexpressed in Glioma Tissues, and Its Inhibition Highly Increases Late Apoptosis in U87MG Cells With Low-density Specificity**. *In vivo (Athens, Greece)* 2022, **36**(2):694-703.

26. Zhang Z, Deng X, Liu Y, Liu Y, Sun L, Chen F: **PKM2, function and expression and regulation**. *Cell & bioscience* 2019, **9**:52.

27. Gao J, Zhao Y, Li T, Gan X, Yu H: **The Role of PKM2 in the Regulation of Mitochondrial Function: Focus on Mitochondrial Metabolism, Oxidative Stress, Dynamic, and Apoptosis. PKM2 in Mitochondrial Function**. *Oxidative medicine and cellular longevity* 2022, **2022**:7702681.

28. Zong Y, Feng S, Cheng J, Yu C, Lu G: **Up-Regulated ATF4 Expression Increases Cell Sensitivity to Apoptosis in Response to Radiation**. *Cellular physiology and biochemistry : international journal of experimental cellular physiology, biochemistry, and pharmacology* 2017, **41**(2):784-794.

29. Wortel IMN, van der Meer LT, Kilberg MS, van Leeuwen FN: **Surviving Stress: Modulation of ATF4-Mediated Stress Responses in Normal and Malignant Cells**. *Trends in endocrinology and metabolism: TEM* 2017, **28**(11):794-806.

30. Fan CL, Yao ZH, Ye MN, Fu LL, Zhu GN, Dai Y, Yao XS: **Fuziline alleviates isoproterenol-induced myocardial injury by inhibiting ROS-triggered endoplasmic reticulum stress via PERK/eIF2α/ATF4/Chop pathway**. *Journal of cellular and molecular medicine* 2020, **24**(2):1332-1344.

31. Huang W, Qiu S, Tong X, He Y, Wang Z, Dong Y: **Activating transcription factor 4 is required for high glucose inhibits proliferation and differentiation of MC3T3-E1 cells**. *Journal of receptor and signal transduction research* 2019, **39**(5-6):407-414.

32. Wang C, Huang Z, Du Y, Cheng Y, Chen S, Guo F: **ATF4 regulates lipid metabolism and thermogenesis**. *Cell research* 2010, **20**(2):174-184.

33. Seo J, Fortuno ES, 3rd, Suh JM, Stenesen D, Tang W, Parks EJ, Adams CM, Townes T, Graff JM: **Atf4 regulates obesity, glucose homeostasis, and energy expenditure**. *Diabetes* 2009, **58**(11):2565-2573.

34. Chen HW, Liu MQ, Zhang GZ, Zhang CY, Wang ZH, Lin AX, Kang JH, Liu WZ, Guo XD, Wang YD *et al*: **Proanthocyanidins inhibit the apoptosis and aging of nucleus pulposus cells through the PI3K/Akt pathway delaying intervertebral disc degeneration**. *Connective tissue research* 2022:1-13.

35. Smith CO, Eliseev RA: **Energy Metabolism During Osteogenic Differentiation: The Role of Akt**. *Stem cells and development* 2021, **30**(3):149-162.

36. Zeng Z, Jing D, Zhang X, Duan Y, Xue F: **Cyclic mechanical stretch promotes energy metabolism in osteoblast-like cells through an mTOR signaling-associated mechanism**. *International journal of molecular medicine* 2015, **36**(4):947-956.

37. Li X, Wang X, Hu Z, Chen Z, Li H, Liu X, Yong ZY, Wang S, Wei Z, Han Y *et al*: **Possible involvement of the oxLDL/LOX-1 system in the pathogenesis and progression of human intervertebral disc degeneration or herniation**. *Scientific reports* 2017, **7**(1):7403.

38. Illien-Jünger S, Gantenbein-Ritter B, Grad S, Lezuo P, Ferguson SJ, Alini M, Ito K: **The combined effects of limited nutrition and high-frequency loading on intervertebral discs with endplates**. *Spine* 2010, **35**(19):1744-1752.

39. Li X, Yang S, Han L, Mao K, Yang S: **Ciliary IFT80 is essential for intervertebral disc development and maintenance**. *Faseb j* 2020, **34**(5):6741-6756.

40. Chang H, Cai F, Zhang Y, Xue M, Liu L, Yang A, Liu X: **Early-stage autophagy protects nucleus pulposus cells from glucose deprivation-induced degeneration via the p-eIF2α/ATF4 pathway**. *Biomedicine & pharmacotherapy = Biomedecine & pharmacotherapie* 2017, **89**:529-535.

41. Liu Y: **Hydrogen peroxide induces nucleus pulposus cell apoptosis by ATF4/CHOP signaling pathway**. *Experimental and therapeutic medicine* 2020, **20**(4):3244-3252.

42. Wang HJ, Hsieh YJ, Cheng WC, Lin CP, Lin YS, Yang SF, Chen CC, Izumiya Y, Yu JS, Kung HJ *et al*: **JMJD5 regulates PKM2 nuclear translocation and reprograms HIF-1α-mediated glucose metabolism**. *Proceedings of the National Academy of Sciences of the United States of America* 2014, **111**(1):279-284.

43. Luo W, Hu H, Chang R, Zhong J, Knabel M, O'Meally R, Cole RN, Pandey A, Semenza GL: **Pyruvate kinase M2 is a PHD3-stimulated coactivator for hypoxia-inducible factor 1**. *Cell* 2011, **145**(5):732-744.

44. Ye J, Mancuso A, Tong X, Ward PS, Fan J, Rabinowitz JD, Thompson CB: **Pyruvate kinase M2 promotes de novo serine synthesis to sustain mTORC1 activity and cell proliferation**. *Proceedings of the National Academy of Sciences of the United States of America* 2012, **109**(18):6904-6909.

45. Schoepflin ZR, Silagi ES, Shapiro IM, Risbud MV: **PHD3 is a transcriptional coactivator of HIF-1α in nucleus pulposus cells independent of the PKM2-JMJD5 axis**. *FASEB journal : official publication of the Federation of American Societies for Experimental Biology* 2017, **31**(9):3831-3847.

46. Johansen JL, Sager TN, Lotharius J, Witten L, Mørk A, Egebjerg J, Thirstrup K: **HIF prolyl hydroxylase inhibition increases cell viability and potentiates dopamine release in dopaminergic cells**. *Journal of neurochemistry* 2010, **115**(1):209-219.

47. Zhang X, Hu Z, Hao J, Shen J: **Low Intensity Pulsed Ultrasound Promotes the Extracellular Matrix Synthesis of Degenerative Human Nucleus Pulposus Cells Through FAK/PI3K/Akt Pathway**. *Spine* 2016, **41**(5):E248-254.

48. Lo WC, Chiou CS, Tsai FC, Chan CH, Mao S, Deng YH, Wu CY, Peng BY, Deng WP: **Platelet-Derived Biomaterials Inhibit Nicotine-Induced Intervertebral Disc Degeneration Through Regulating IGF-1/AKT/IRS-1 Signaling Axis**. *Cell transplantation* 2021, **30**:9636897211045319.

49. Yang Y, Wang X, Liu Z, Xiao X, Hu W, Sun Z: **Osteogenic protein-1 attenuates nucleus pulposus cell apoptosis through activating the PI3K/Akt/mTOR pathway in a hyperosmotic culture**. *Bioscience reports* 2018, **38**(6).

50. Zhao Y, Li A: **miR-19b-3p relieves intervertebral disc degeneration through modulating PTEN/PI3K/Akt/mTOR signaling pathway**. *Aging* 2021, **13**(18):22459-22473.

51. Cui A, Ding D, Li Y: **Regulation of Hepatic Metabolism and Cell Growth by the ATF/CREB Family of Transcription Factors**. *Diabetes* 2021, **70**(3):653-664.

52. Riscal R, Schrepfer E, Arena G, Cissé MY, Bellvert F, Heuillet M, Rambow F, Bonneil E, Sabourdy F, Vincent C *et al*: **Chromatin-Bound MDM2 Regulates Serine Metabolism and Redox Homeostasis Independently of p53**. *Molecular cell* 2016, **62**(6):890-902.

53. Ganapathy-Kanniappan S, Kunjithapatham R, Geschwind JF: **Anticancer efficacy of the metabolic blocker 3-bromopyruvate: specific molecular targeting**. *Anticancer research* 2013, **33**(1):13-20.

54. He S, Jia Q, Zhou L, Wang Z, Li M: **SIRT5 is involved in the proliferation and metastasis of breast cancer by promoting aerobic glycolysis**. *Pathology, research and practice* 2022, **235**:153943.

**Figure legend**

Figure 1 Nutritional deficiency can inhibit increase the expression of ATF4 and inhibit PKM2 in NP cells

(A) The NP cell proliferation status detected by CCK8 under the different condition of nutritional deficiency (B)The expression level of ATF4 and PKM2 detected by western blot under the different condition of nutritional deficiency. (Group A, B, C, D denotes the media contain 10%, 5%, 1% and 0% FBS, respectively) (C) The quantity analysis of expression level of ATF4 and PKM2 as showed in B (D) The expression level of ATF4 and PKM2 detected by western blot under serum free condition with different time (E) The quantity analysis of expression level of ATF4 and PKM2 as showed in D. Data are mean ± s.d. The percentage calculation in each tissue were measured at least 1000 cells each sample. Statistical significance was determined by one-way ANOVA and Student's t-test. **, ##, △△ P < .001.

Figure 2 ATF4 silence can induce NP cell’s apoptosis under the nutritional deficiency condition

(A)ATF4 expression level detected by RT-qPCR (B) ATF4 expression level detected by western blot (C) The quantity analysis of expression level of ATF4 as showed in B (D) The NP cell proliferation status detected by CCK8 under the condition of nutritional deficiency and ATF4 silence (E)The cell apoptosis status detected by flow cytometry under the condition of nutritional deficiency and ATF4 silence (F) The quantity data showed percentage of cells in apoptosis as showed in E (G) The cell apoptosis protein detected by western blot (Group A, B, C denotes the 10%FBS, 1%FBS and 1%FBS with siATF4 group respectively) (H) The quantity analysis of expression level of cell apoptosis protein as showed in G. Data are mean ± s.d The percentage calculation in each tissue were measured at least 1000 cells each sample. Statistical significance was determined by one-way ANOVA and Student's t-test. **, ##, △△ P < .001.

Figure 3 ATF4 silence can increase NP cell’s glucose uptake and lactic acid level under the nutritional deficiency condition

(A)The NP cell’s glucose uptake level under the condition of nutritional deficiency and ATF4 silence (Group A, B, C denotes the 10%FBS, 1%FBS and 1%FBS with siATF4 group respectively) (B) The NP cell’s lactic acid level under the condition of nutritional deficiency and ATF4 silence (Group A, B, C denotes the 10%FBS, 1%FBS and 1%FBS with siATF4 group respectively) (C) The glucolysis status detected by western blot under the condition of nutritional deficiency and ATF4 silence (Group A, B, C denotes the 10%FBS, 1%FBS and 1%FBS with siATF4 group respectively) (D) The quantity the protein reflected glucolysis status as showed in C. Data are mean ± s.d. The percentage calculation in each tissue were measured at least 1000 cells each sample. Statistical significance was determined by one-way ANOVA and Student's t-test. **, ##, △△ P < .001.

Figure 4 ATF4 silence can induce NP cell’s apoptosis under the nutritional deficiency condition may via promoting AKT phosphate

(A) The AKT and P-AKT expression level detected by western blot under the condition of nutritional deficiency and ATF4 silence (Group A, B, C denotes the 10%FBS, 1%FBS and 1%FBS with siATF4 group respectively) (B) The quantity analysis of expression level of cell apoptosis protein as showed in A. Data are mean ± s.d. The percentage calculation in each tissue were measured at least 1000 cells each sample. Statistical significance was determined by one-way ANOVA and Student's t-test. **, ##, △△ P < .001.

Figure 5 PKM2 inhibition can reduce the cell apoptosis induced by ATF4 silence under the nutritional deficiency condition

(A) The NP cell proliferation status detected by CCK8 induced by ATF4 silence or PKM2 inhibition under the nutritional deficiency condition (B) The cell apoptosis status detected by flow cytometry under the condition of nutritional deficiency and ATF4 silence or PKM2 inhibition (C) The quantity data showed percentage of cells in apoptosis as showed in B. Data are mean ± s.d. The percentage calculation in each tissue were measured at least 1000 cells each sample. Statistical significance was determined by one-way ANOVA and Student's t-test. **, ##, △△ P < .001.

Figure 6. PKM2 inhibition can reduce NP cell’s glucose uptake and lactic acid level induced by ATF4 silence under the nutritional deficiency condition

(A) The NP cell’s glucose uptake level induced by ATF4 silence or PKM2 inhibition under the nutritional deficiency condition (B) The NP cell’s lactic acid level induced by ATF4 silence or PKM2 inhibition under the nutritional deficiency condition (C) The glucolysis status detected by western blot induced by ATF4 silence or PKM2 inhibition under the nutritional deficiency condition (Group A, B, C, D, E denotes the 10%FBS, 1%FBS, 1%FBS with siATF4, 1%FBS with PKM2-IN-1, 1%FBS+siATF4+ PKM2-IN-1 group respectively) (D) The quantity the protein reflected glucolysis status as showed in C. Data are mean ± s.d. The percentage calculation in each tissue were measured at least 1000 cells each sample. Statistical significance was determined by one-way ANOVA and Student's t-test. **, ##, △△ P < .001.

Figure 7 PKM2 inhibition reduce the cell apoptosis induced by ATF4 silence under the nutritional deficiency condition by inhibiting AKT phosphate

(A) The expression level of cell apoptosis protein detected by western blot induced by ATF4 silence or PKM2 inhibition under the nutritional deficiency condition (Group A, B, C, D, E denotes the 10%FBS, 1%FBS, 1%FBS with siATF4, 1%FBS with PKM2-IN-1, 1%FBS+siATF4+ PKM2-IN-1 group respectively) (B) The quantity analysis of expression level of cell apoptosis protein as showed in A (C)The AKT and P-AKT expression level detected by western blot induced by ATF4 silence or PKM2 inhibition under the nutritional deficiency condition (Group A, B, C, D, E denotes the 10%FBS, 1%FBS, 1%FBS with siATF4, 1%FBS with PKM2-IN-1, 1%FBS+siATF4+ PKM2-IN-1 group respectively) (D) The quantity analysis of expression level of cell apoptosis protein as showed in C. Data are mean ± s.d. The percentage calculation in each tissue were measured at least 1000 cells each sample. Statistical significance was determined by one-way ANOVA and Student's t-test. **, ##, △△ P < .001.
