# Supplementary material for: Nutritional deficiency induces nucleus pulposus cell apoptosis via the ATF4-PKM2-AKT signal ﻿axis
Source: BMC Musculoskelet Disord. 2022 Nov 2;23:946. doi: 10.1186/s12891-022-05853-1 (PMC9628105; doi:10.1186/s12891-022-05853-1)
Supplement: Supplementary file 2 — Supplementary Material 2 [file 12891_2022_5853_MOESM2_ESM.pdf]

Figure 1B

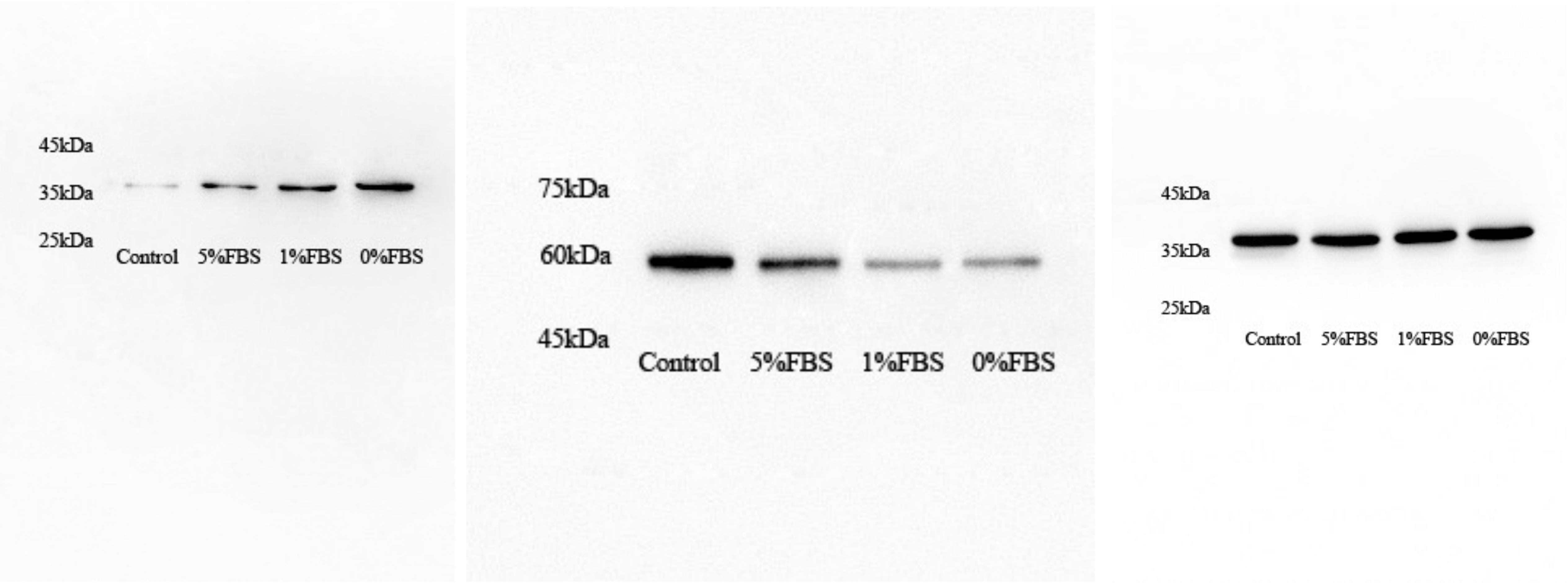

ATF4

PKM2

GAPDH

Figure 1D

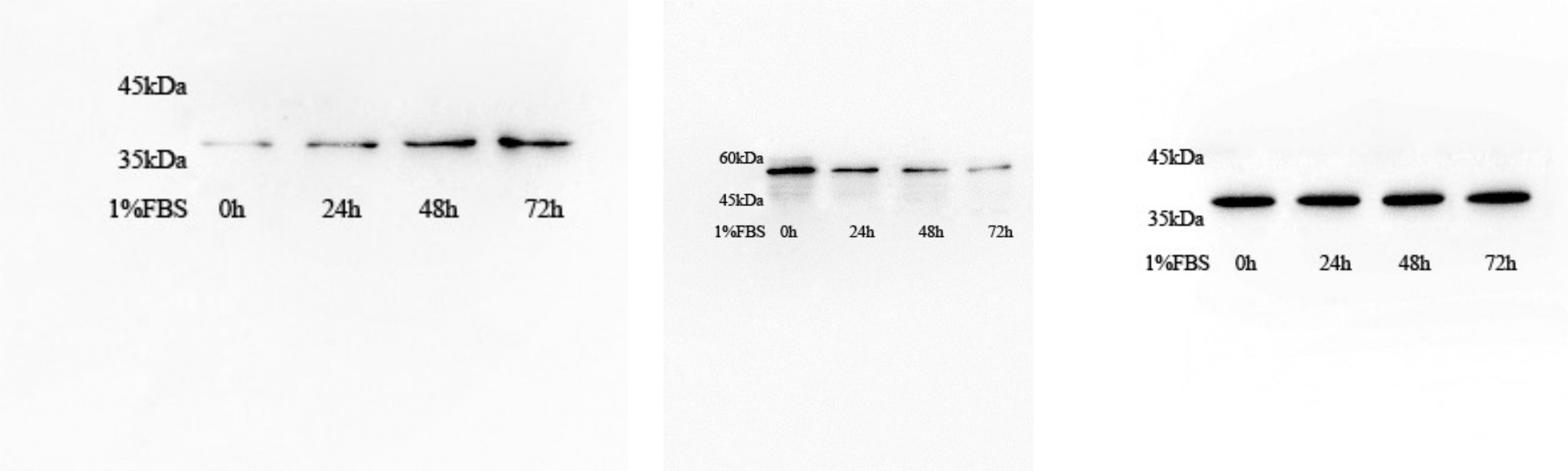

ATF4 PKM2 GAPDH

Figure 2B

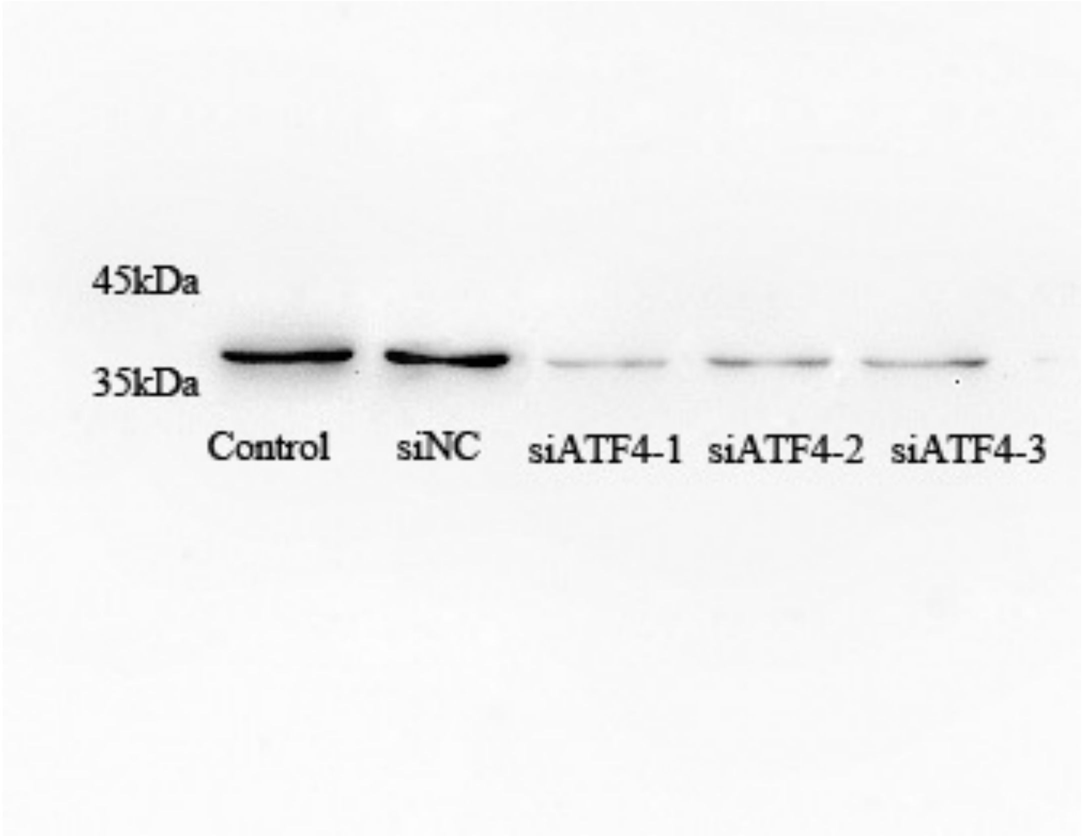

ATF4

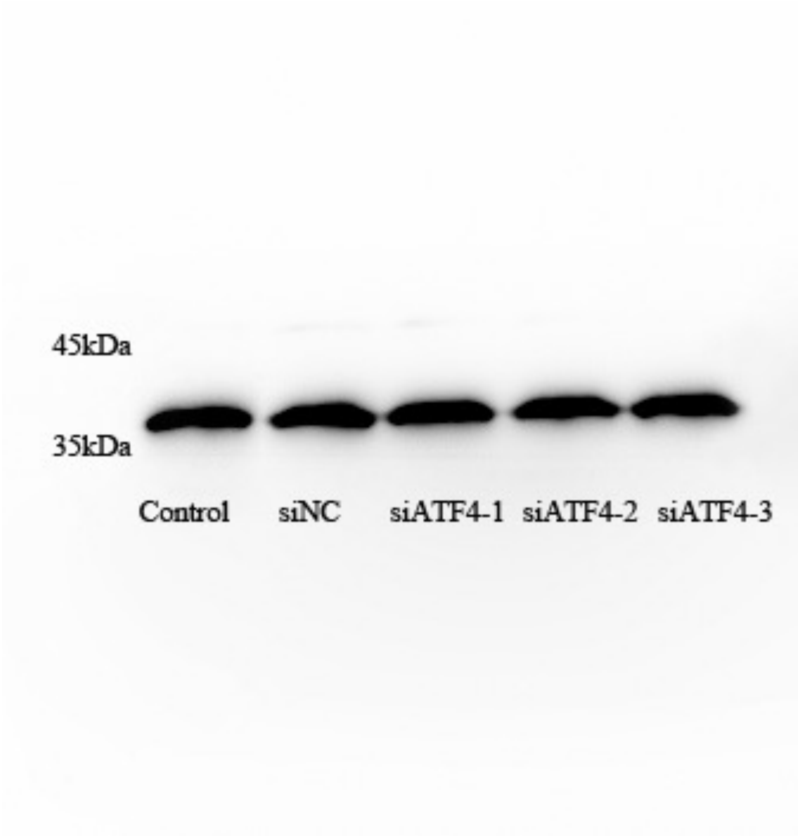

GAPDH

Figure 2G

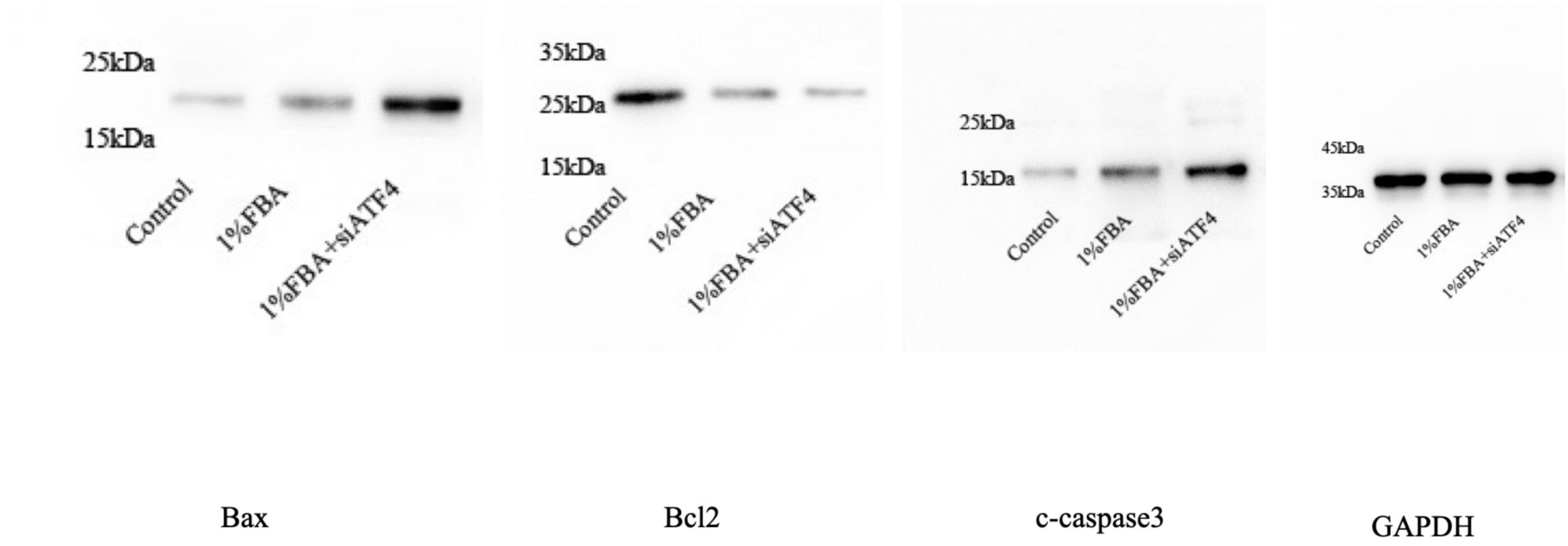
25kDa
15kDa
Control
1%FBA
1%FBA+siATF4
35kDa
25kDa
15kDa
Control
1%FBA
1%FBA+siATF4
25kDa
15kDa
Control
1%FBA
1%FBA+siATF4
45kDa
35kDa
Control
1%FBA
1%FBA+siATF4
Bax
Bcl2
c-caspase3
GAPDH

Figure 3C

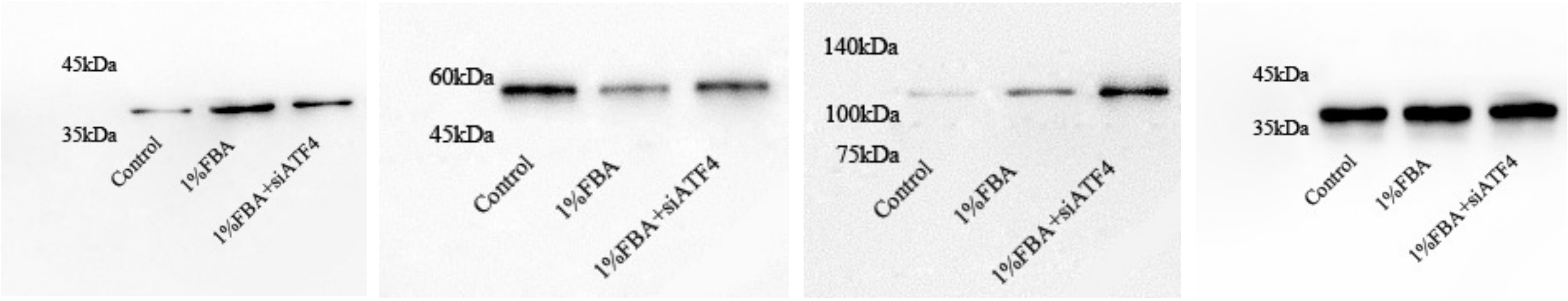

ATF4 PKM2 HIF GAPDH

Figure 4A

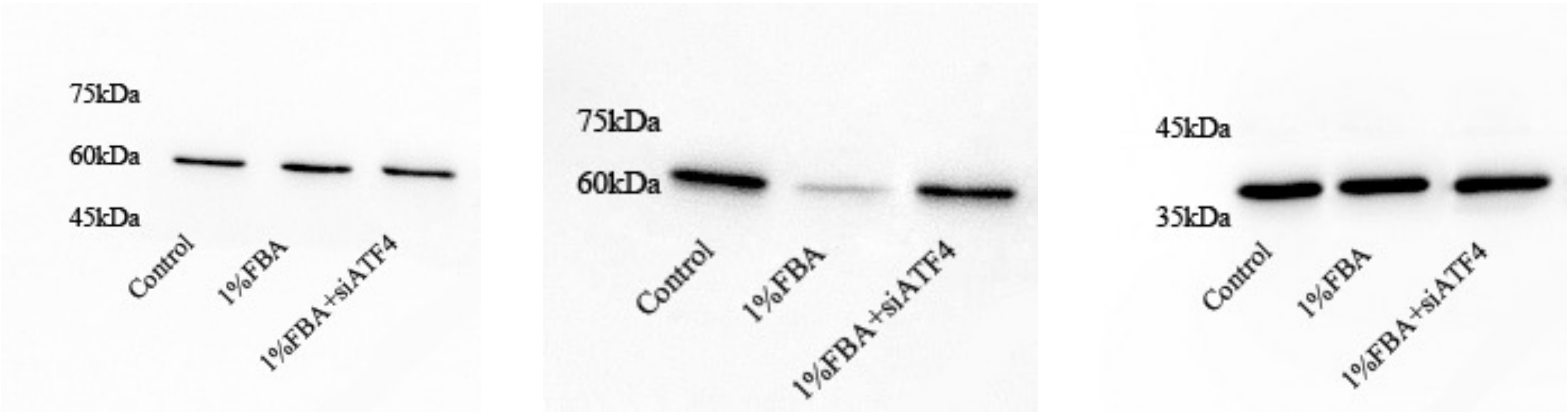

AKT p-AKT GAPDH

Figure 6C

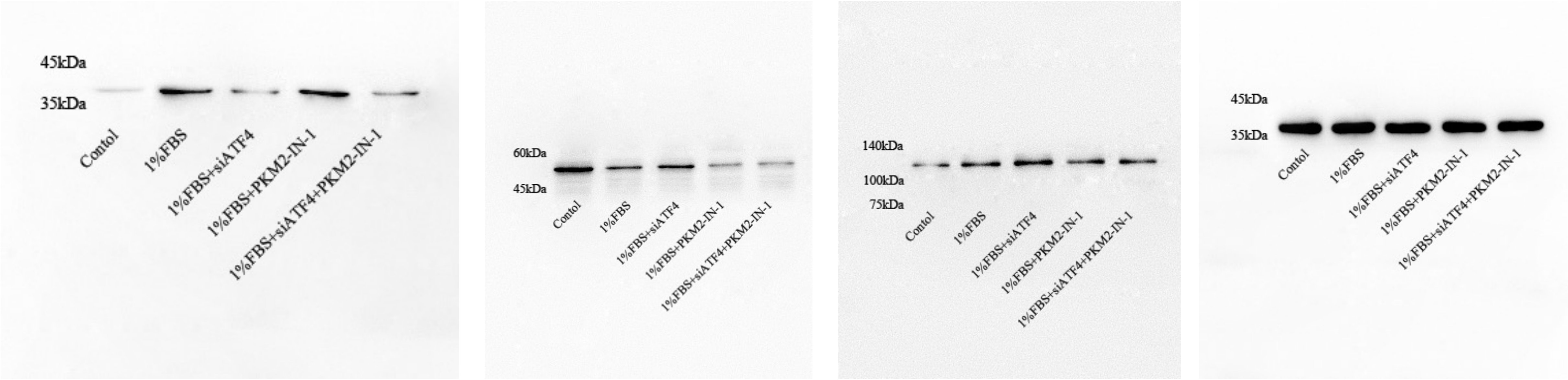

ATF4 PKM2 HIF GAPDH

Figure 7A

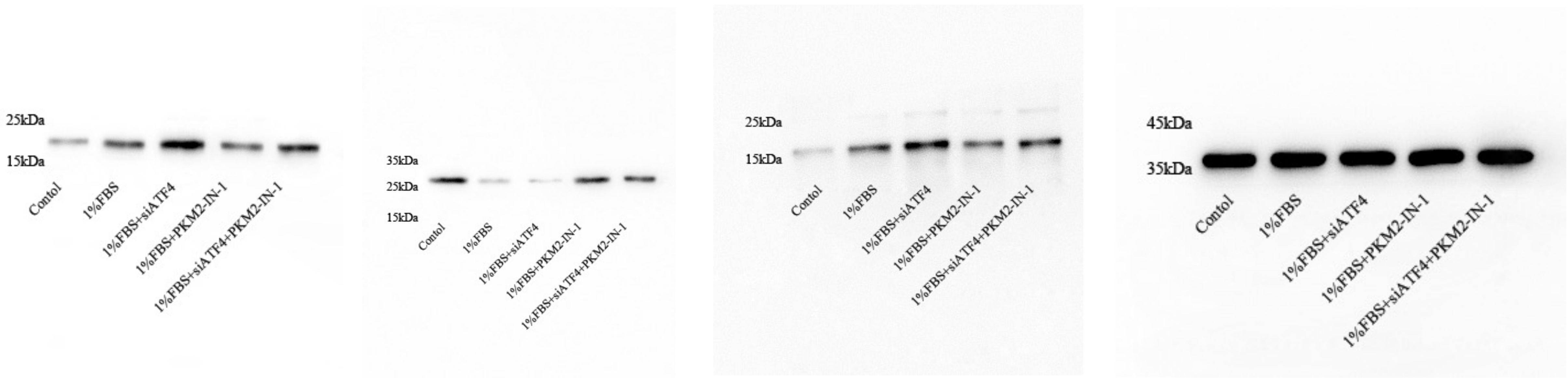

Bax Bcl2 c-caspase3 GAPDH

Figure 7C

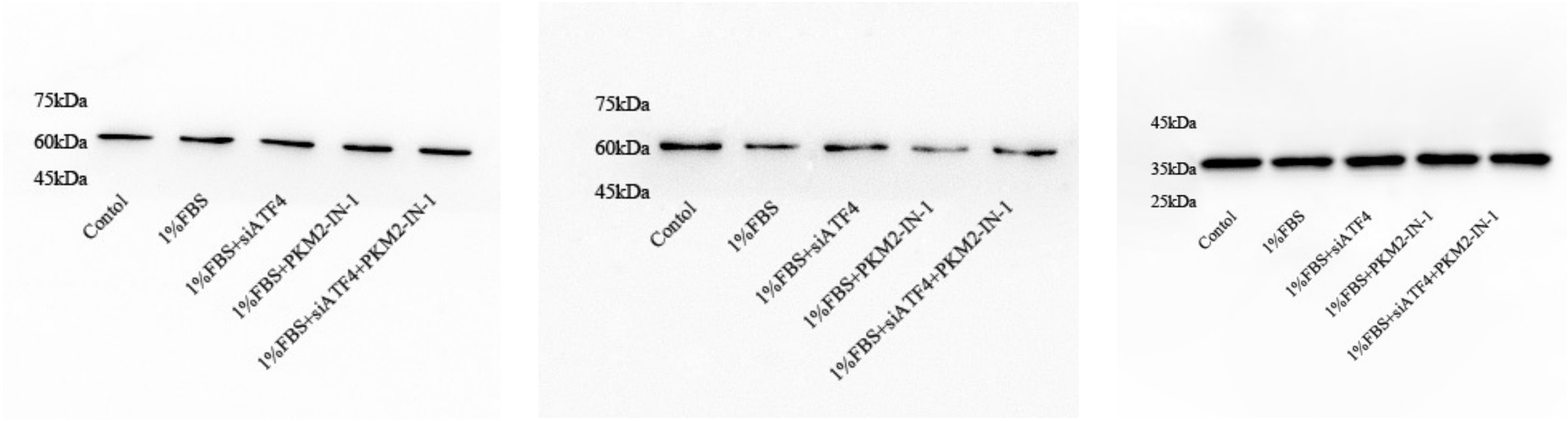

AKT p-AKT GAPDH
