# Supplementary material for: Nutritional deficiency induces nucleus pulposus cell apoptosis via the ATF4-PKM2-AKT signal ﻿axis
Source: BMC Musculoskelet Disord. 2022 Nov 2;23:946. doi: 10.1186/s12891-022-05853-1 (PMC9628105; doi:10.1186/s12891-022-05853-1)
Supplement: Supplementary file 3 — Supplementary Material 3 [file 12891_2022_5853_MOESM3_ESM.pdf]

Figure 1B

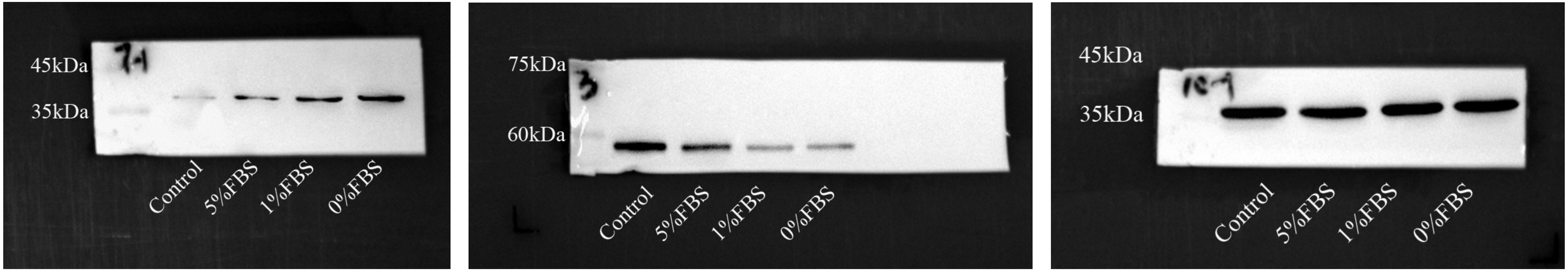

ATF4+39kDa PKM2+58kDa GAPDH+37kDa

Figure 1D

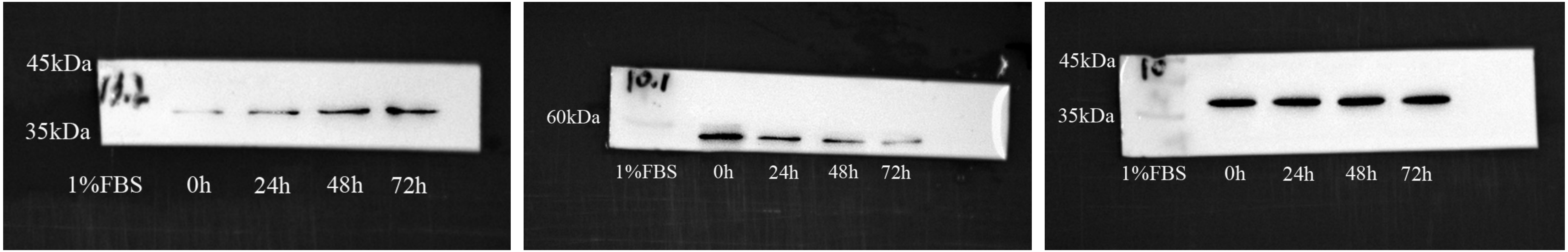

ATF4+39kDa PKM2+58kDa GAPDH+37kDa

Figure 2B

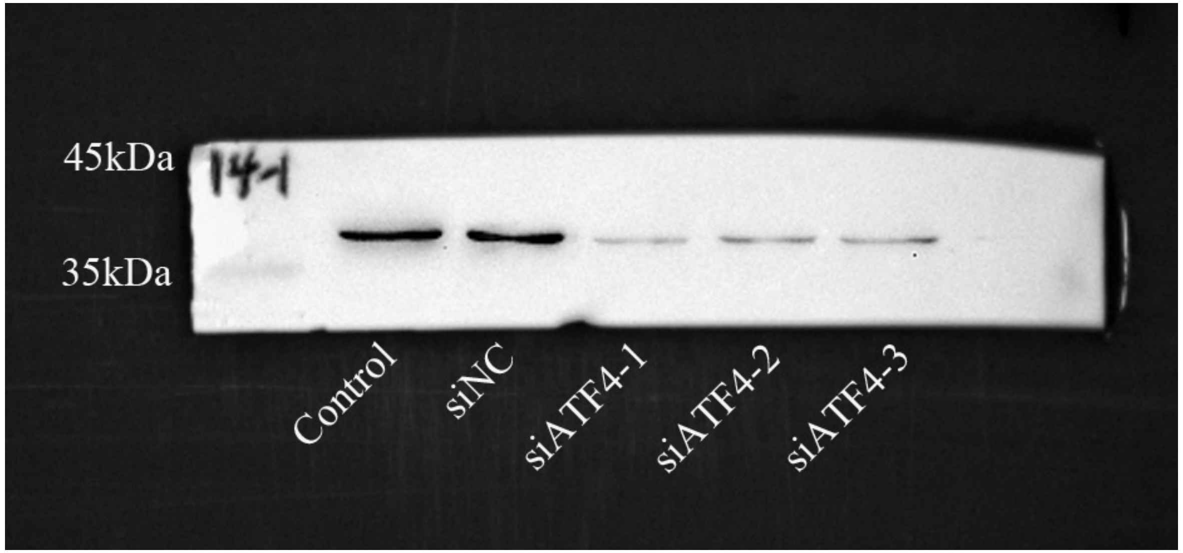

ATF4+39kDa

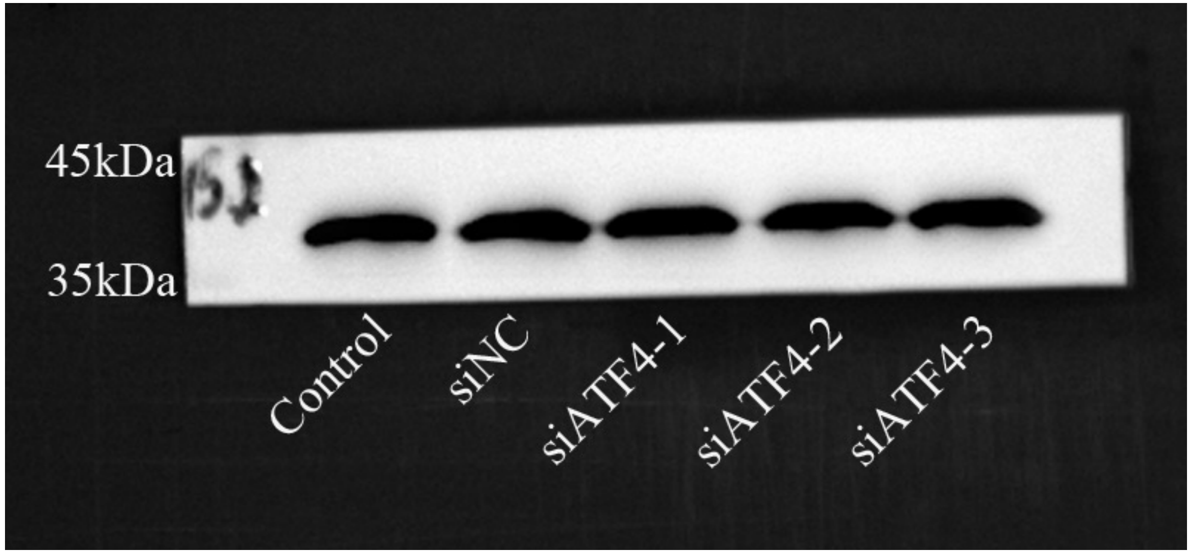

GAPDH+37kDa

Figure 2G

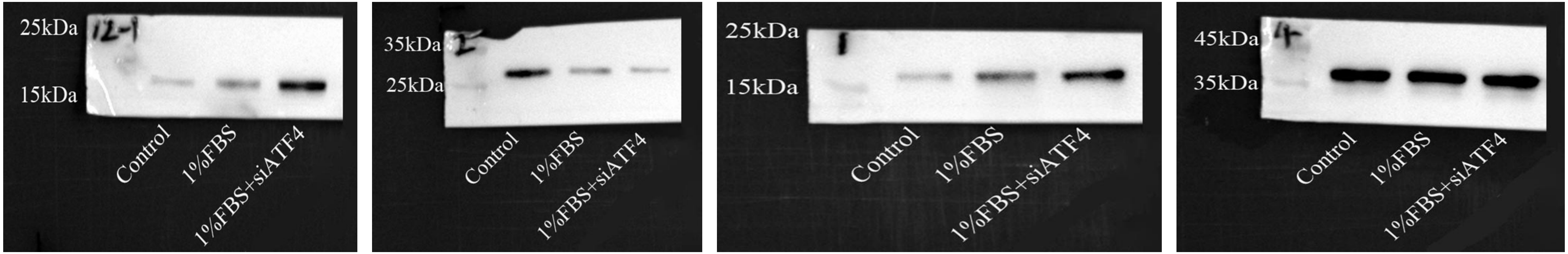

Bax+21kDa

Bcl2+26kDa

C-caspsse-3+17kDa

GAPDH+37kDa

Figure 3C

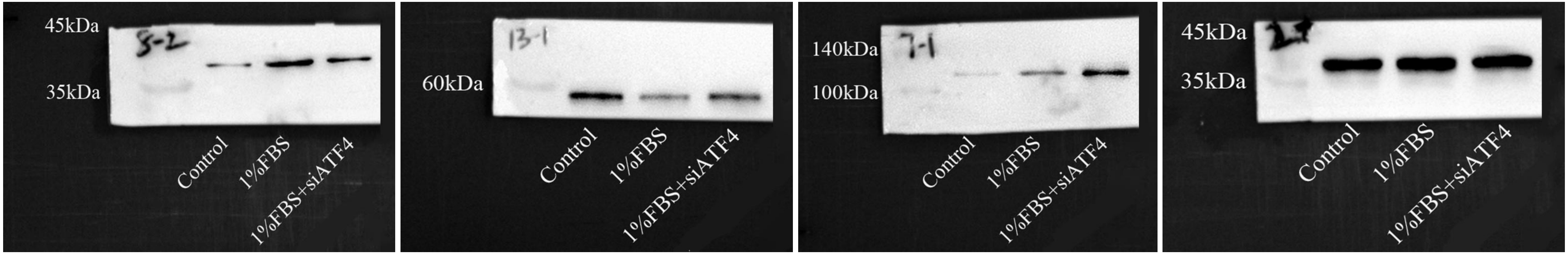

ATF4+39kDa PKM2+58kDa HIF-1a+120kDa GAPDH2+37kDa

Figure 4A

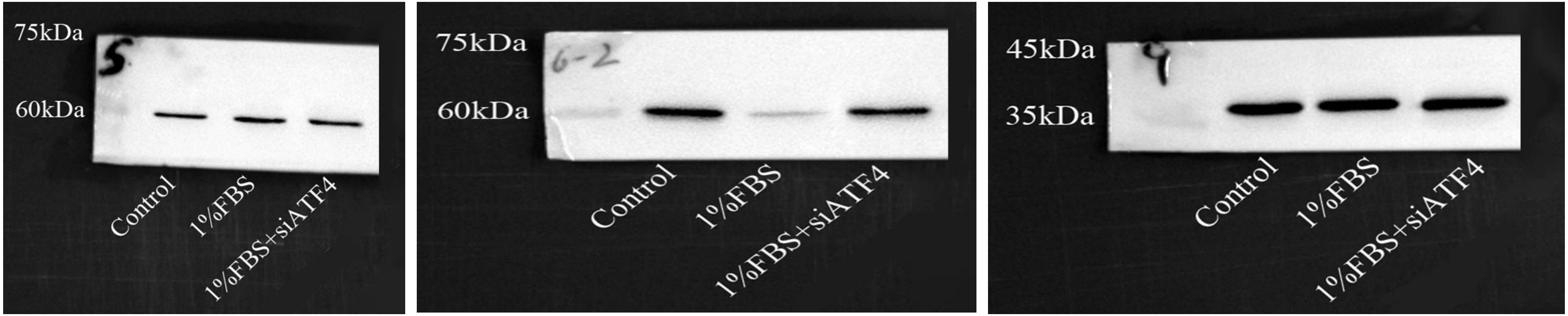

AKT+60kDa

p-AKT+60kDa

GAPDH+37kDa

Figure 6C

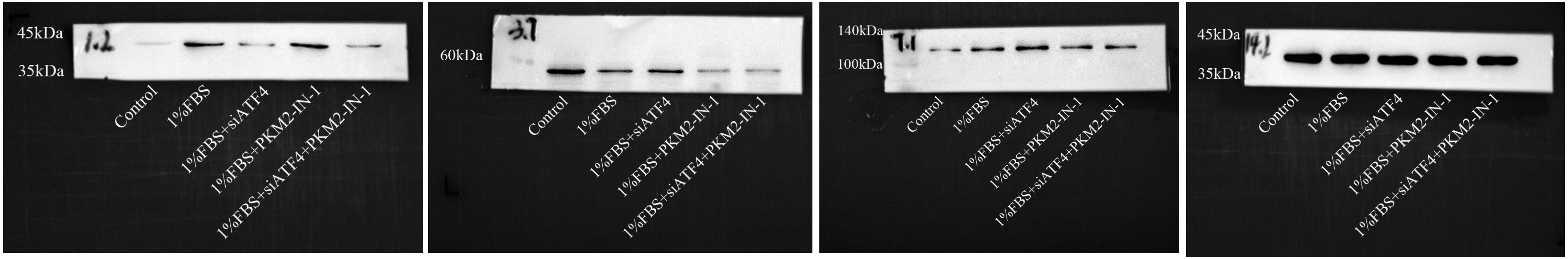

ATF4+39kDa PKM2+58kDa HIF-1a+120kDa GAPDH+37kDa

Figure 7A

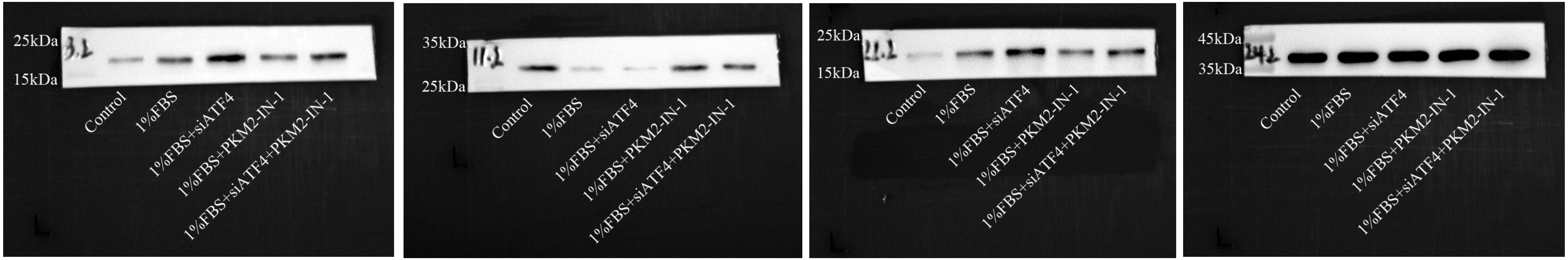

Bax+21kDa

Bcl2+26kDa

C-caspase3+17kDa

GAPDH+37kDa

Figure 7C

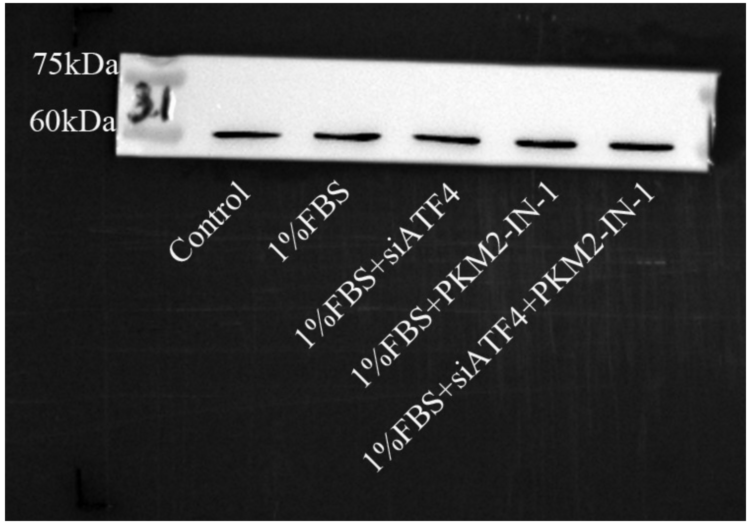

AKT+60kDa

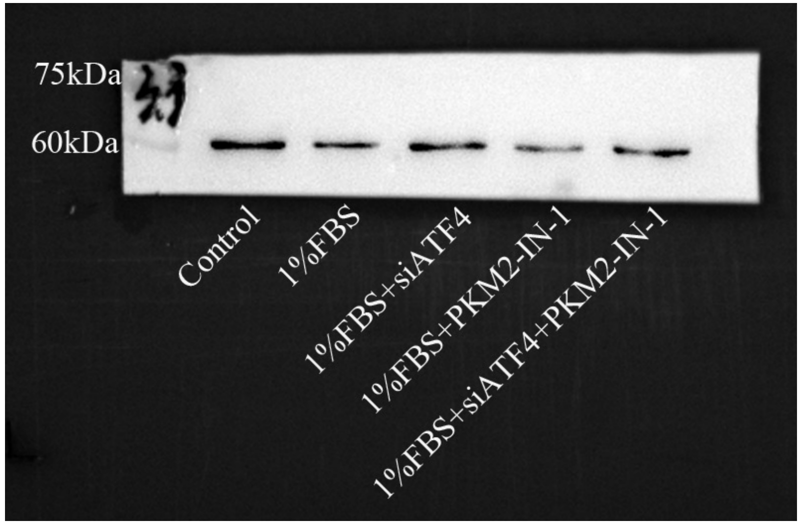

p-AKT+60kDa

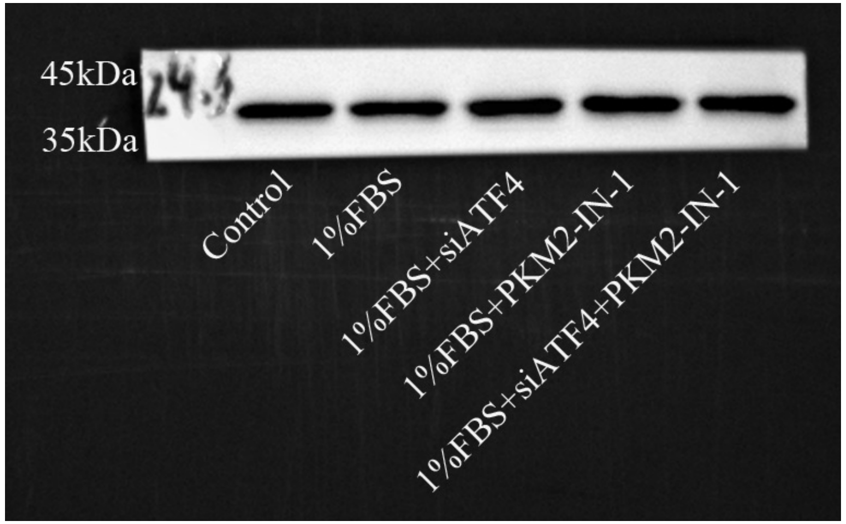

GAPDH+37kDa
